# Comment on “Strong signature of the active Sun in 100 years of terrestrial insolation data” by W. Weber

**DOI:** 10.1002/andp.201100179

**Published:** 2011-09-20

**Authors:** Georg Feulner

**Affiliations:** Earth System Analysis, Potsdam Institute for Climate Impact Research (PIK)P.O. Box 60 12 03, 14412 Potsdam, Germany

**Keywords:** Climate, solar activity, solar irradiance, cosmic rays

## Abstract

An analysis of ground-based observations of solar irradiance was recently published in this journal, reporting an apparent increase of solar irradiance on the ground of the order of 1% between solar minima and maxima [1]. Since the corresponding variations in total solar irradiance on top of the atmosphere are accurately determined from satellite observations to be of the order of 0.1% only [2], the one order of magnitude stronger effect in the terrestrial insolation data was interpreted as evidence for cosmic-ray induced aerosol formation in the atmosphere. In my opinion, however, this result does not reflect reality. Using the energy budget of Earth's surface, I show that changes of ground-based insolation with the solar cycle of the order of 1% between solar minima and maxima would result in large surface air temperature variations which are inconsistent with the instrumental record. It would appear that the strong variations of terrestrial irradiance found by [1] are due to the uncorrected effects of volcanic or local aerosols and seasonal variations. Taking these effects into account, I find a variation of terrestrial insolation with solar activity which is of the same order as the one measured from space, bringing the surface energy budget into agreement with the solar signal detected in temperature data.

## 1 Earth's surface energy budget

Using a simple argument based on the energy budget at Earth's surface one can show that a variation of terrestrial insolation with solar activity on the percentage level – as reported in [1] – would lead to unrealistically large temperature fluctuations which are not observed in the instrumental surface temperature record. To first order, the relationship between surface temperature *T*_s_ and terrestrial insolation *I*_s_ is governed by the balance between the absorbed short-wave radiation *I*_s_(1 – α_s_) and the long-wave emission 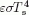 according to the Stefan-Boltzmann law, with the surface reflectivity or albedo α_s_, the emissivity ε and the Stefan-Boltzmann constant σ. Therefore, changes in temperature *dT* are related to changes in insolation *dI* by


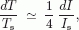
(1)

since changes of surface albedo can be neglected for such small irradiance variations. Conventional wisdom suggests that terrestrial insolation *I*_s_ varies with the solar cycle in the same way as the total solar irradiance (TSI) above the atmosphere, i.e. *dI/I*_s_ ≃ 0.1% [2]. Using this value and an average surface temperature of *T*_s_ = 288 K yields an expected temperature variation over the solar cycle of *dT* ≃ 0.07 K. This simple approximation ignores feedbacks in the climate system (due to clouds, water vapour, ice and changes in the lapse rate). The combination of these effects is generally considered to act as a positive feedback, the estimate above can thus be considered a lower limit.

A more appropriate estimate for the temperature response *dT* to changes in solar irradiance *dI* including these feedbacks is given by the relation


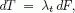
(2)

with the change in radiative forcing *dF* = (1 − α)*dI*/4 (the change *dI* in incoming solar radiation *I* ≃ 1361 W m^−2^ corrected for Earth's albedo α ≃ 0.3 and geometry) and the transient climate sensitivity λ_*t*_ ≃ 0.4 K/(W m^−2^) [3] describing the short-term response of the climate system to changes. For a change of 0.1% in insolation this yields *dT* ≃ 0.1 K, in excellent agreement with the value derived from global surface air temperature data [4,5].

**Fig. 1:**
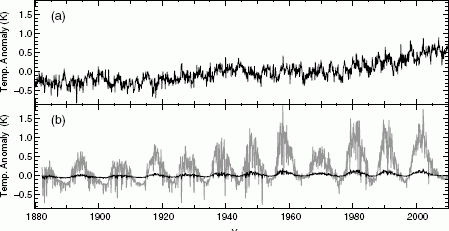
(a) Monthly values for the global surface air temperature anomaly (relative to the average 1951–1980) [7]. Other global surface temperature datasets look very similar. (b) Monthly temperature anomalies due to the 11-year solar cycle according to [4] *(black line)* on the same scale as panel (a). The *grey line* indicates the response expected for a ten-times larger variation in terrestrial irradiance as suggested by [1] which is inconsistent with the temperature record shown in panel (a).

This estimate is interesting for two reasons: First, it shows that most of the observed solar-cycle global temperature variation can be explained by changes in top-of-the-atmosphere irradiance alone. The remainder is likely due to well documented effects of changes in ultraviolet radiation, leaving little room for more speculative effects of cosmic rays. Secondly, a terrestrial insolation variation with solar activity one magnitude larger than the TSI changes (as suggested by the results in [1]) would lead to global temperature changes of *dT* ≃ 1 K between solar maxima and minima (Fig. 1b), a result clearly in conflict with the instrumental surface air temperature record (e.g. [6]) which does not exhibit such large variations over the 11-year solar cycle (see Fig. 1a).

This discrepancy between the strong variation of terrestrial insolation reported in [1] and the energy budget at Earth's surface suggests that the data analysis in [1] is biased by systematic effects not related to solar activity changes, a hypothesis which will be explored in the next section.

## 2 Analysis of the terrestrial insolation data

The analysis of ground-based insolation data in [1] neglects the effects of atmospheric aerosols from volcanoes or local pollution and seasonal variations, which in my opinion feigns a stronger influence of solar activity on terrestrial insolation. These arguments will be briefly summarised in this section, the complete re-analysis of the data is discussed in detail in [8].

**Fig. 2:**
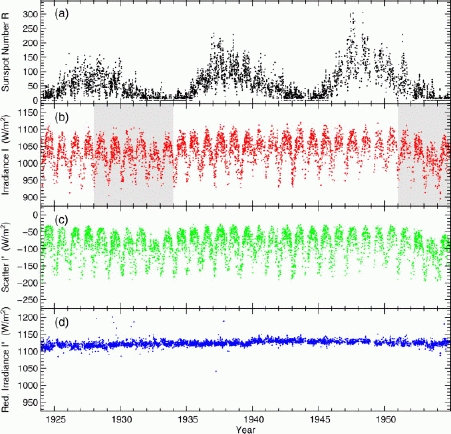
(online colour at: http://www.ann-phys.org) (a) Daily sunspot numbers [11] for the time from 1924 to 1955. (b) Daily values of terrestrial solar irradiance as measured at Cerro Montezuma at airmass 2 during the same time period. Grey-shading indicates periods affected by volcanic aerosols or local pollution [10]. (c) Scatter function of the irradiance. (d) Reduced irradiance, i.e. the irradiance with the scatter function subtracted to correct for variations in atmospheric water and aerosol content.

The bias resulting from volcanic aerosols and pollution is illustrated in Fig. 2. During the time period 1924–1955 covered by the Smithsonian Astrophysical Observatory (SAO) data [9] analysed in [1], the years 1928–1934 and 1951–1955 were affected by aerosols from well documented volcanic eruptions and local pollution [10]. By coincidence, these periods overlap with two out of three of the solar minima during this interval (Fig. 2a). Since aerosols absorb and scatter incoming light, this results in a lower measured irradiance on the ground, as can be seen in Fig. 2b. Note that there is no apparent drop in irradiance during the minimum around 1944 which is unaffected by aerosols, demonstrating that the lower irradiance during the other two solar minima is indeed driven by aerosols rather than the low solar activity. (Note that the expected variation of the irradiance with solar activity of the order of 0.1 % is too small to be seen with the naked eye on this scale.)

In his reply [12] to this comment, Werner Weber observes that the scatter in the derived TSI during these periods of active volcanism is not larger than at other times and concludes that the SAO personnel did not take observations during these time intervals. This is not entirely convincing, however, since the effects of volcanic aerosols are visible for a few years after the eruptions, and there clearly exist data during these time periods. I would argue that the small scatter in the TSI demonstrates that the SAO method for correcting the observed irradiance for the measured effects of water vapour and aerosols even work at times of large volcanic aerosol loads in the atmosphere.

The seasonal bias in the data is a bit more intricate. Terrestrial irradiance exhibits a strong seasonal cycle (see Fig. 2b). Since days with low and high sunspot numbers are not distributed equally over all seasons in the SAO data, an analysis of trends of irradiance with sunspot number will be skewed if the seasonal variations are not corrected [8].

A reanalysis of the data corrected for seasonal variations and without the years affected by volcanic or other aerosols yields a trend of irradiance with sunspot number which is about a factor of ten smaller than the one reported in [1], corresponding to a variation of 0.1% between solar maxima and minima as for the TSI [8]. This variation of terrestrial insolation is in agreement with the temperature changes associated with the solar cycle [4,5], see the energy estimate presented in Sect. 1.

Since the publication of the original results in [1], Werner Weber has introduced an improved analysis technique for the SAO data described in his reply to this comment [12]. Starting with the observation that the measured terrestrial irradiance shows a second-order dependence on the precipitable water content *W* and the brightness *A* of the solar aureole, a ‘scatter function’ *I*′ can be constructed by approximating a second-order power series in *W* and *A* to the observed irradiance (see Fig. 2c for an example). Subtracting this scatter function *I*′ from the irradiance measurements *I* yields the ‘reduced irradiance’ *I*″ which exhibits a drastically reduced scatter as compared to the original irradiance (see Fig. 2d). This method is quite remarkable and is a legitimate technique to remove the effects of seasons and volcanic aerosols; physically it is equivalent to correcting the observed terrestrial irradiance for the effects of water vapour and aerosols in the atmosphere using empirical data.

In addition, however, Werner Weber argues that the scatter function *I*′ depends strongly on the sunspot number *R* and that the *R* dependence of *I*′ has to be removed by applying a Legendre-type transformation *I*′ → **I**′ with 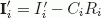. The variation of *I*′, however, is more likely caused by the effects of aerosols rather than by solar activity (see the discussion above and in [8]). Hence by applying the transformation an unrealistic *R* dependence of the reduced irradiance *I*″ is introduced in [12], leading to an artificially large variation of the terrestrial irradiance with sunspot number and a striking, yet misleading correlation between sunspot number and strong variations in the reduced (and transformed) terrestrial irradiance shown in Fig. 4 in [12].

This effect is best illustrated by the apparent dip in the reduced terrestrial irradiance around the solar minimum in 1945 in [12]: Looking at the original data, there is no decrease in terrestrial insolation and no increase in water content or aureole brightness during that time (see Fig. 2 in [12] and Fig. 2c in [8]), yet Fig. 2 in [12] shows an increase in the transformed scatter function **I**′ and a corresponding dip in the reduced irradiance **I**″ = *I* − **I**′. These features can be naturally explained as artefacts of the transformation based on the assumption that the scatter function *I*′ depends on sunspot number *R*. Nevertheless, the technique of scatter reduction (without the transformation) introduced in [12] might offer the possibility to improve the analysis of terrestrial irradiance trends provided that the effects of solar activity and volcanism can be reliably separated. Indeed, the trends of the reduced irradiance (un-transformed) with sunspot number are in agreement with the results found in [8] and thus with the surface energy balance discussed in Sect. 1.

## 3 Discussion: Solar activity and Earth's climate

Quantifying the influence of solar activity on Earth's climate [13] is clearly an important issue, helping to distinguish between natural and anthropogenic causes of climate change. The energy balance of Earth's surface (Sect. 1) and investigations of the instrumental temperature record [4,5] show, however, that the temperature changes caused by the 11-year solar cycle amount to 0.1 K only, much smaller than the observed 20th-century warming of about 0.7 K [6]. On longer timescales, extended periods of low solar activity (‘grand minima’) like the 17th-century Maunder Minimum [14] are associated with a global cooling of only a few tenths of a degree [15], although regional and seasonal temperature signatures are more pronounced. Furthermore, solar activity appears not to be the dominant cause of the ‘Little Ice Age” coinciding with the Maunder Minimum. Both lower greenhouse gas concentrations and strong volcanic eruptions during the 17th century contributed to the observed cooling [16,17]. This also means that even a future grand solar minimum could not offset the much larger temperature rise caused by anthropogenic greenhouse-gas emissions [18,19].
